# A complete and multifaceted overview of antibiotic use and infection diagnosis in the intensive care unit: results from a prospective four-year registration

**DOI:** 10.1186/s13054-018-2178-7

**Published:** 2018-09-29

**Authors:** Liesbet De Bus, Bram Gadeyne, Johan Steen, Jerina Boelens, Geert Claeys, Dominique Benoit, Jan De Waele, Johan Decruyenaere, Pieter Depuydt

**Affiliations:** 1 Department of Critical Care Medicine, Ghent University Hospital, C. Heymanslaan 10, 9000 Ghent, Belgium; 2 Department of Laboratory Medicine, Ghent University Hospital, C. Heymanslaan 10, 9000 Ghent, Belgium; 3 Heymans Institute of Pharmacology, Ghent University, C. Heymanslaan 10, 9000 Ghent, Belgium

**Keywords:** Antibiotic stewardship, Intensive care unit, Electronic surveillance, Longitudinal surveillance, Infection

## Abstract

**Background:**

Preparing an antibiotic stewardship program requires detailed information on overall antibiotic use, prescription indication and ecology. However, longitudinal data of this kind are scarce. Computerization of the patient chart has offered the potential to collect complete data of high resolution. To gain insight in our global antibiotic use, we aimed to explore antibiotic prescription in our intensive care unit (ICU) from various angles over a prolonged time period.

**Methods:**

We studied all adult patients admitted to Ghent University Hospital ICU from 1 January 2013 until 31 December 2016. Antibiotic prescription data were prospectively merged with diagnostic (suspected focus, severity and probability of infection at the time of prescription, or prophylaxis) and microbiology data by ICU physicians during daily workflow through dedicated software. Definite focus of infection and probability of infection (classified as high/moderate/low) were reassessed by dedicated ICU physicians at patient discharge.

**Results:**

During the study period, 8763 patients were admitted and overall antibiotic consumption amounted to 1232 days of therapy (DOT)/1000 patient days. Antibacterial DOT (84% of total DOT) were linked with infection in 80%; the predominant foci were the respiratory tract (49%) and the abdomen (19%). A microbial cause was identified in 56% (3169/5686). Moderate/low probability infections accounted for 42% of antibacterial DOT prescribed for respiratory tract infections; for abdominal infections, this figure was 15%. The median treatment duration of moderate/low probability respiratory infections was 4 days (IQR 3–7). Antifungal DOT (16% of total DOT) were linked with infection in 47% of total antifungal DOT. Antifungal prophylaxis was primarily administered in the surgical ICU (76%), with a median duration of 4 DOT (IQR 2–9).

**Conclusions:**

By prospectively combining antibiotic, microbiology and clinical data we were able to construct a longitudinal, multifaceted dataset on antibiotic use and infection diagnosis. A complete overview of this kind may allow the identification of antibiotic prescription patterns that require future antibiotic stewardship attention.

**Electronic supplementary material:**

The online version of this article (10.1186/s13054-018-2178-7) contains supplementary material, which is available to authorized users.

## Background

Antibiotics are among the most prescribed drugs in the intensive care unit (ICU) [1, 2]. The concept of “antibiotic stewardship” refers to policies and interventions to optimize antibiotic therapy and restrict their unnecessary use [3–9]. The latter comprises avoiding antibiotic prescription for non-infectious disease, limiting the use of broad-spectrum drugs when a narrower antimicrobial spectrum suffices and shortening duration of therapy when prolonged antibiotic courses do not provide benefit [10–12].

Surveillance of antibiotic prescription is a first and essential step to measure antibiotic expenditure, to document physicians’ incentives to prescribe antibiotics and to identify areas of potential overuse or misuse which could then be a target for antimicrobial stewardship interventions [5, 13–15]. In general, surveillance metrics are derived from antibiotic prescription data (pharmacy-based), microbiology results (laboratory-based) or diagnostic codes (administration-based) or a combination thereof. However, surveillance is not the primary purpose of these sources of information and using them often results in poor matching of antibiotic prescription data with the corresponding clinical and microbiological information. As such, their ability to represent the complex nature of the antibiotic treatment decision-making process and thus their practical usefulness is limited [16–18]. Prospective surveillance is more precise and informative but, since it is demanding in time and resources, is usually only applied for relatively short periods of time or for a limited scope of prescription, e.g. for certain classes of reserved antibiotics [19]. However, the computerization of the patient ICU chart has nowadays offered the potential to record healthcare processes as complete data of high resolution in a way that minimally interferes with the healthcare deliverer’s workflow [20].

In this manuscript, we present a complete and in-depth analysis of global antibiotic prescription and infection diagnosis in a university hospital ICU over a 4-year period. These data were collected with the help of a locally developed software program, which has been designed to link pharmaceutical, clinical and microbiological data together with diagnostic interpretation while performing daily bedside clinical work. As such, we were able to get a bird’s eye view of our local antibiotic prescribing practices, which can then serve as a starting point for the future construction of an antibiotic stewardship program (ASP).

## Methods

### Setting

This study was conducted from 1 January 2013 until 31 December 2016 at the medical (14 beds) and surgical (22 beds) ICU of Ghent University Hospital (1054 beds). The Ghent University Hospital Ethics Committee approved the study (registration number B670201628197) and waived informed consent based on the non-interventional nature of this study and the complete anonymization of patient data. Patients aged 16 years or older were included.

An Intensive Care Information System (Centricity Critical Care, GE Health Care) integrating computerized physician order entry for medication prescriptions, computerized medication administration recording and clinical patient monitoring data has been available at the bedside since 2003. Patients are managed in a closed ICU model. Antibiotic prescriptions are at the discretion of the attending senior ICU physician, without the use of stringent protocols or antibiotic restrictions. As a rule, postoperative prophylactic treatment is not prolonged for more than 24 h following the procedure. Empirical antibiotic choices are guided by systematically collected surveillance cultures whenever available. Direct microscopic examination is performed on all diagnostic respiratory and per-operative samples. Pathogen identification is routinely performed by matrix-assisted laser desorption ionization time-of-flight mass spectrometry (MALDI-TOF MS). Microbiology results are reported electronically. Interdisciplinary staff meetings with medical microbiologists reviewing all antibiotic prescriptions take place once weekly in the medical ICU and three times weekly in the surgical ICU; these staff also include the presence of infectious diseases specialists in the surgical ICU. In addition, daily advice and follow up by these specialties is possible on a demand basis. Treatment duration and opportunities for antibiotic de-escalation are evaluated daily by the attending ICU physician and during the interdisciplinary discussions.

A ventilation-associated pneumonia (VAP) prevention bundle was used during the entire study period and involved the use of strict hand hygiene, oral care with chlorhexidine, endotracheal tube cuff pressure control (between 20 and 30 cm of H_2_O), a semi-recumbent position (30–45°) and daily assessment of sedation.

A software program with the acronym COSARA (Computer-based Surveillance and Alerting of infections, antimicrobial Resistance and Antibiotic consumption in the ICU) was developed by a consortium of the Ghent University Hospital ICU and the Department of Information Technology (INTEC) of the Faculty of Engineering of Ghent University [20]. The project was funded by the Flemish government. The software has been fully operational since 2010 at the study ICU and since then its use has become incorporated into routine daily patient care (e.g. during ward rounds and interdisciplinary staff meetings). The goal of COSARA is to support the physician in the daily workflow by automatically integrating all relevant infection-related data (clinical parameters, antibiotic prescription, laboratory variables including microbiology and chest x-ray images) from different data sources and presenting these as a graphic overview (Additional files 1, 2, 3 and 4). The validity of COSARA as a surveillance tool and the feasibility of continued infection registration through the software has been described in a preceding study [21]. With the help of COSARA, all antibiotic prescriptions are prospectively labeled: diagnostic (suspected focus, severity and probability of infection, or prophylaxis) and microbiological information is first entered into the system by ICU physicians at the time of antibiotic prescription, which is then definitively reassessed by dedicated ICU physicians (LDB and PD) at patient discharge. Probability is classified as low, moderate or high, as described previously [21], using clinical, radiological and microbiological criteria.

Prescription indication and antimicrobial utilization were described for all patients that were admitted during this study period. In microbiologically confirmed infections, the timing of appropriate antibiotic therapy was defined as the point in time at which all pathogens involved in the infection were covered by at least one component of the treatment.

Antimicrobial days of therapy (DOT) per admission and per patient days is recommended as utilization metric by the STEWARDS panel and others [13, 14, 22]. In agreement with the recommendations of the Centers for Disease Control and Prevention - National Healthcare Safety Network (CDC-NHSN), DOT is defined as the number of days with systemic administration of at least one dose of an antimicrobial agent as recorded by COSARA [12].

Subgroup analyses were performed in the patient populations with an ICU length of stay (LOS) equal to or more than 48 h versus less than 48 h, respectively, as this latter subgroup consists mainly of the less severely ill patients in our setting (e.g. patients who are postoperative, have minor trauma or are being monitored). In addition, patients with an ICU LOS of 48 h or more constitute the population at risk of developing ICU-acquired infection, which was defined as an infection emerging after more than 48 h of ICU admission.

### Statistics

Categorical variables were expressed as frequencies (percentages) and continuous variables were described as medians with the interquartile range (IQR; 25–75th percentile). Differences in categorical variables were calculated using the Pearson chi-square test. The Mann–Whitney U test was used to compare continuous variables. Statistical significance was defined as *p* < 0.05. For outcome analysis, we only included the last ICU episode of patients with consecutive ICU admissions. Statistical analysis was performed using R Statistical Software (version 3.4.2).

## Results

### Patients

A total of 10,743 ICU admissions were recorded in 8763 patients, resulting in a total of 47,403 patient days from 1 January 2013 until 31 December 2016. ICU and hospital mortality were 10.7% and 15%, respectively. Median Acute Physiology and Chronic Health Evaluation (APACHE) II score at admission was 18 (IQR 13–25). Mechanical ventilation was provided in 3958 admissions (36.8%) with a median duration of 2 days (IQR 1–6), resulting in 20,897 ventilation days. Vasopressor therapy was administered in 3639 admissions (33.9%) with a median duration of 2 days (IQR 2–4). Detailed information on patient characteristics is presented in Additional file 5.

Methicillin resistance was present in 23% of the *Staphylococcus aureus* isolates in our ICU population. Vancomycin resistance was present in 1.9% of the *Enterococcus* species isolates. Extended spectrum beta-lactamase production (ESBL) was present in 33% of *Enterobacteriaceae* isolates, whereas carbapenemase production was present in 1.2%.

**Fig. 1:**
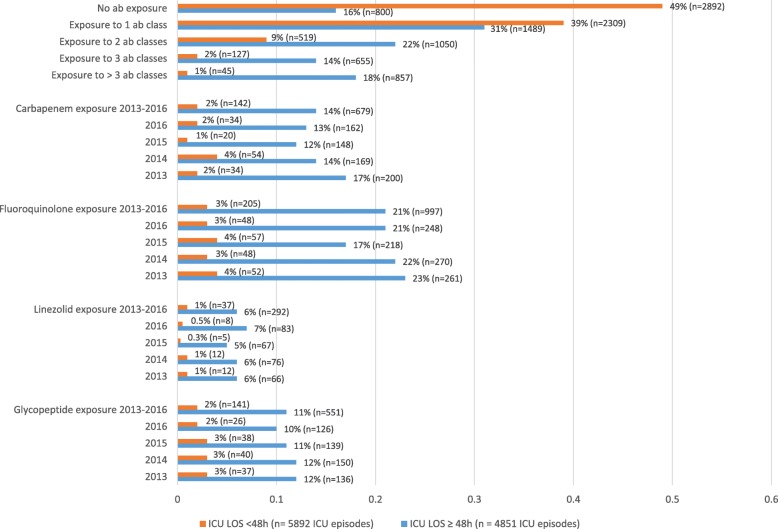
Antibiotic exposure per ICU episode. ab, antibiotic; ICU, intensive care unit; LOS, length of stay

Patients were exposed to at least one antibiotic class in 66% (7051/10743) of ICU admissions. An infection was present within the first 48 h of ICU admission in 35% (3804/10743) of admissions. An ICU-acquired infection was diagnosed in 23% (1096/4851) of admissions with an ICU length of stay of more than 48 h. Detailed information on antibiotic exposure per ICU episode is provided in Fig. 1.

### Prescription indication

**Fig. 2:**
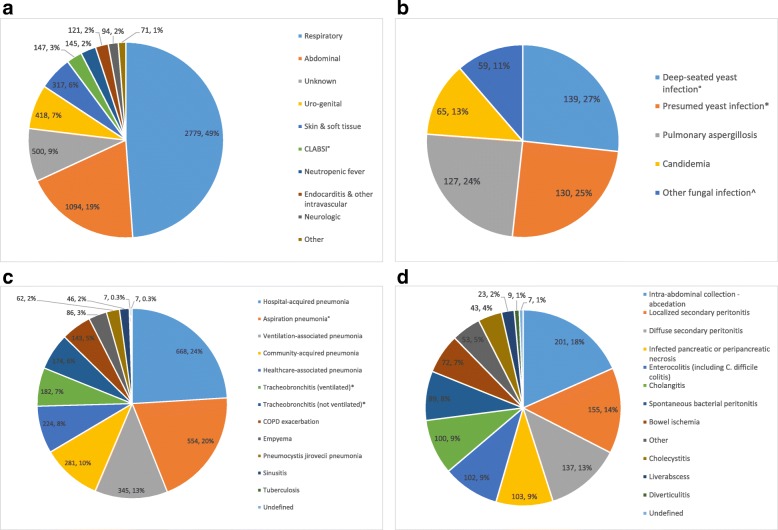
Bacterial and fungal infection focus. **a** Focus of bacterial infections (*n* = 5686); infection probability was classified as low, moderate or high in 14%, 27% and 59% of the bacterial infections, respectively; CLABSI, central-line-associated bloodstream infection. CLABSI incidence was 3.8/1000 catheter days. Crude ICU mortality rate in patients with CLABSI was 11.4%. **b** Focus of fungal infections (*n* = 520); infection probability was classified as low, moderate or high in 12%, 17% and 71% of the fungal infections, respectively; °presence of yeast in a normally sterile body site combined with clinical signs of infection; *fungal infection considered clinically likely by treating physician in the absence of yeast in a normally sterile body site; ^mucocutaneous candidiasis, candidiasis of the genitourinary tract, extra-pulmonary *Aspergillus* infection, invasive non-*Aspergillus* mold infection. **c** Bacterial respiratory infection (*n* = 2779); °bacterial pneumonia following macroaspiration; *tracheobronchitis criteria include fever, purulent tracheobronchial secretions, isolation of a respiratory pathogen of a good quality lower respiratory tract sample, no radiographic signs of new pneumonia. **d** Bacterial abdominal infection (*n* = 1094)

A total number of 10,731 treatment courses (infection bars) was recorded during the study period. Respectively 4525 (42.2%) and 6206 (57.8%) of these courses were prescribed for prophylaxis and for infection. Fungal infections represented 8% (520/6206) of the infectious episodes. Infections were microbiologically confirmed in 56% (3169/5686) and 63% (327/520) of bacterial and fungal infections, respectively. Antibiotic treatment initiated in the ICU was considered appropriate within 12 h and 24 h, respectively, following treatment initiation in 83% and 87% of microbiologically confirmed infections. Infections were ICU-acquired in 28% of cases (1767/6206). The focus of the bacterial infections was predominantly respiratory and abdominal (respectively, 49% and 19%). The crude ICU mortality rate in patients with respiratory and abdominal infections was 22.1% and 19.2%, respectively. Infection probability was classified as high, moderate or low in 50%, 34% and 16%, respectively, of the respiratory infections compared to 76%, 16% and 8%, respectively, of the abdominal infections. The median treatment duration of high, moderate and low probability respiratory infections for which the course was completed on the ICU was 7 days (IQR 5–10), 6 days (IQR 4–8)] and 4 days (IQR 2–6), respectively. Only 19% of the respiratory infections were classified as ventilator-associated. A total of 345 cases of VAP and 182 cases of ventilator-associated tracheobronchitis (VAT) were diagnosed, resulting in VAP and VAT incidences of 16.5/1000 ventilation days and 8.7/1000 ventilation days, respectively. Respectively, 52%, 37% and 11% of VAP were classified as having a high, moderate or low probability of infection, translating into VAP incidences of 8.6, 6.1 and 1.8/1000 ventilation days, respectively. VAP incidence was 17.0/1000 ventilation days in the medical ICU and 16.1/1000 ventilation days in the surgical ICU. The median treatment duration of VAP and VAT episodes in the ICU was 7 days (IQR 5–9) and 6 days (IQR 4–7), respectively. The crude ICU mortality rate in patients with VAP and VAT was 32.6% and 21.1%, respectively (see Fig. 2 for more details on bacterial and fungal infection focus). Additional file 6 contains more details on treatment duration per infection focus.

### Antimicrobial utilization

A total of 14,908 antibiotic courses (antibiotic bars) were administered, resulting in 58,413 DOT (1232 DOT/1000 patient days). Detailed utilization analysis per antibiotic agent and infection probability is presented in Table 1. Utilization analysis per antibiotic agent per year is presented in Additional file 7. Anti-pseudomonal penicillins combined with a beta-lactamase inhibitor, non-anti-pseudomonal penicillins combined with a beta-lactamase inhibitor and fluoroquinolones were the most frequently used classes of antibiotics (respectively, 218 DOT/1000 patient days, 172 DOT/1000 patient days and 114 DOT/1000 patient days); azoles were the predominantly used class of antifungal drug (162 DOT/1000 patient days).

**Table 1:** Antimicrobial utilization per antimicrobial class and per infection probability

	DOT (%)	DOT/1000 patient days	DOT (% of total DOT/antibiotic class)			
			Infection present			Prophylactic treatment
			High probability	Moderate probability	Low probability	
Antibacterial class						
Aminoglycosides	474 (1.0)	10.0	388 (81.8)	67 (14.1)	5 (1.1)	6 (1.3)
Ansamycins (rifampicin)	268 (0.5)	5.7	230 (85.8)	9 (3.4)	13 (4.9)	15 (5.6)
Carbapenems	4488 (9.1)	94.7	3438 (76.6)	697 (15.5)	221 (4.9)	110 (2.5)
1st gen. cephalosporins	2939 (6.0)	62.0	–	–	–	2939 (100)
2nd gen. cephalosporins	1192 (2.4)	25.1	398 (33.4)	324 (27.2)	169 (14.2)	301 (25.3)
3rd gen. cephalosporins	1955 (4.0)	41.2	1343 (68.7)	400 (20.5)	143 (7.3)	63 (3.2)
Fluoroquinolones	5385 (11)	113.6	3268 (60.7)	1285 (23.9)	367 (6.8)	448 (8.3)
Folate pathway inhibitor	3105 (6.3)	65.5	896 (28.9)	319 (10.3)	142 (4.6)	1747 (56.3)
Glycopeptides	2966 (6.0)	62.6	2163 (72.9)	438 (14.8)	172 (5.8)	169 (5.7)
Glycylcyclines	319 (0.6)	6.7	242 (75.9)	63 (19.7)	11 (3.4)	2 (0.6)
Lincosamides	806 (1.6)	17.0	564 (70.0)	140 (17.4)	42 (5.2)	60 (7.4)
Macrolides	1421 (2.9)	30.0	834 (58.7)	215 (15.1)	84 (5.9)	284 (20.0)
Monobactams	150 (0.3)	3.2	33 (22.0)	28 (18.7)	10 (6.7)	79 (52.7)
Nitrofurans	59 (0.1)	1.2	19 (32.2)	16 (27.1)	7 (11.9)	17 (28.8)
Nitroimidazoles	1289 (2.6)	27.2	976 (75.7)	147 (11.4)	61 (4.7)	92 (7.1)
Oxazolidinones	1780 (3.6)	37.6	1434 (80.6)	212 (11.9)	53 (3.0)	69 (3.9)
Penicillins	1504 (3.1)	31.7	1212 (80.6)	188 (12.5)	86 (5.7)	15 (1.0)
Non-anti-pseudomonal penicillins + beta-lactamase inhibitor	8136 (16.5)	171.6	3267 (40.2)	1605 (19.7)	660 (8.1)	2588 (31.8)
Anti-pseudomonal penicillins + beta-lactamase inhibitor	10,342 (21.0)	218.2	6405 (61.9)	2292 (22.2)	808 (7.8)	800 (7.7)
Phosphonic acids	27 (0.1)	0.6	3 (11.1)	17 (63.0)	6 (22.2)	1 (3.7)
Polymyxins	469 (1.0)	9.9	311 (66.3)	97 (20.7)	19 (4.1)	42 (9.0)
Tetracyclines	95 (0.2)	2.0	82 (86.3)	1 (1.1)	1 (1.1)	11 (11.6)
Total antibacterial	49,169 (100)	1037.3	27,506 (55.9)	8560 (17.4)	3080 (6.3)	9858 (20.0)
Antifungal class						
Azoles	7684 (83.1)	162.1	2123 (27.6)	415 (5.4)	222 (2.9)	4809 (62.6)
Echinocandins	1354 (14.6)	28.6	1022 (75.5)	176 (13.0)	93 (6.9)	56 (4.1)
Polyenes	206 (2.2)	4.3	138 (67.0)	37 (18.0)	29 (14.1)	–
Total antifungal	9244 (100)	195.0	3283 (35.5)	628 (6.8)	344 (3.7)	4865 (52.6)
Total	58,413 (100)	1232.3	30,789 (52.7)	9188 (15.7)	3424 (5.9)	14,723 (25.2)

*DOT* days of therapy, *gen.* generation

Prophylactic therapy accounted for 25% of the total antimicrobial DOT; first-generation cephalosporins, folate pathway inhibitors, monobactams and azoles were predominantly used in this setting. Antibacterial prophylaxis mainly comprised perioperative prophylaxis (46%) and prolonged prophylaxis in the immunosuppressed patient (19%). The median duration of perioperative prophylaxis was 8 h (IQR 8–17). In addition, 9% and 11%, respectively, of antibacterial prophylaxis was initiated in the setting of trauma and following aspiration with respective durations of 3 DOT (IQR 2–4) in trauma patients and 3 DOT (IQR 2–5) in the setting of aspiration. Antifungal prophylaxis accounted for 53% of the total antifungal DOT and was primarily administered in the surgical ICU (76%), with a median duration of 4 DOT (IQR 2–9).

Seventy-five percent of the total antimicrobial DOT was used to treat infections. Of the total amount of DOT used to treat bacterial respiratory infections, 42% was used to treat infections with a moderate and low probability; for bacterial abdominal infections, this figure was 15%.

### Microbiology

**Fig. 3:**
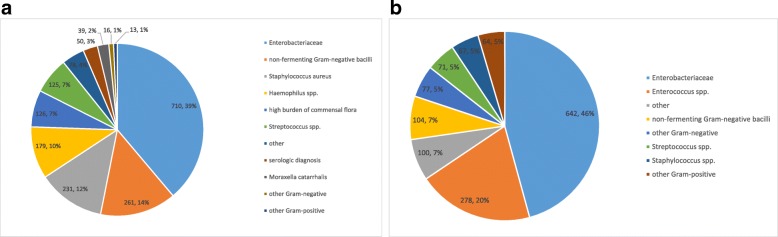
Pathogens linked to bacterial respiratory and abdominal infections. **a** Pathogens linked to bacterial respiratory infection (*n* = 1828); *Enterobacteriaceae* = *Citrobacter* spp., *Enterobacter* spp., *Escherichia coli*, *Hafnia* spp., *Klebsiella* spp., *Morganella* spp., *Proteus* spp., *Providencia* spp., *Serratia* spp. Non-fermenting Gram-negative bacilli = *Achromobacter* spp., *Acinetobacter* spp., *Stenotrophomonas* spp., *Pseudomonas* spp., other non-fermenting Gram-negative bacilli. *Streptococcus* spp. = *Streptococcus pneumoniae*, *Streptococcus pyogenes*, Viridans streptococci, other streptococci. Other = culture results of referral hospital. Serologic diagnosis = *Legionella pneumophila* antigen, *Streptococcus pneumoniae* antigen. **b** Pathogens linked to bacterial abdominal infection (*n* = 1403); *Enterobacteriaceae* = *Citrobacter* spp., *Enterobacter* spp., *Escherichia coli*, *Hafnia* spp., *Klebsiella* spp., *Morganella* spp., *Proteus* spp., *Providencia* spp., *Salmonella* spp., *Serratia* spp., *Yersinia* spp. *Enterococcus* spp. = *Enterococcus faecalis, Enterococcus faecium*, other enterococci. Other = culture results of referral hospital. Non-fermenting Gram-negative bacilli = *Achromobacter* spp., *Stenotrophomonas* spp., *Pseudomonas* spp., other non-fermenting Gram-negative bacilli. *Streptococcus* spp. = *Streptococcus pneumonia*, Viridans streptococci, other streptococci. *Staphylococcus* spp. = *Staphylococcus aureus*, coagulase-negative staphylococci, other. Other Gram-negative = e.g. *Bacteroides* spp., *Prevotella* spp., *Aeromonas* spp., *Campylobacter* spp. Other Gram-positives = e.g. *Clostridium* spp., *Bacillus* spp.

*Enterobacteriaceae* were the predominant bacterial species that were designated as causative pathogens in both respiratory and abdominal infections (respectively, 39% and 46% of all pathogens linked). Amoxicillin/clavulanic acid resistance and cefuroxime resistance was present in 48% and 39% of the Enterobacteriaceae isolates, respectively. Non-fermenting Gram-negative bacilli were the second most prevalent pathogens associated with respiratory infections (15%), and mainly consisted of *Pseudomonas aeruginosa.* Piperacillin-tazobactam, ceftazidime and meropenem resistance was present in 27%, 20% and 20%, respectively, of the *Pseudomonas* isolates. *Enterococci* were the second most prevalent pathogens that were linked as causative in the abdominal infections (20%) and ampicillin resistance was present in 44% of the isolates (Fig. 3).

## Discussion

In this manuscript, we demonstrated the versatility of a detailed database in antibiotic use and infection diagnosis, which is prospectively built by linking prescription, clinical and microbiological data on individual ICU patients during clinical workflow. This allowed various analyses that respectively center on patient admissions, prescription indication and infection diagnosis, antibiotic utilization and microbiology and as such may be useful to support various aspects of infection control and antibiotic stewardship. To the best of our knowledge, our study is the largest single-center study providing epidemiological data on antibiotic consumption and infections treated in the ICU in terms of number of ICU beds (36) and timespan covered (4 years).

Our study confirms that the antibiotic burden in the ICU is very high. Respectively, 66% of all admitted patients and 84% of patients with an ICU stay of more than 48 h were exposed to at least one class of antibiotic. These figures are consistent with the results of the one-year prospective surveillance study of Bergmans et al. and with the EPIC II point-prevalence study, which reported antibiotic prescription in 61% and 71% of admitted patients, respectively [1, 2].

The need for detailed antibiotic prescription surveillance and feedback to the clinician was already acknowledged in the very early stages of antimicrobial stewardship, but there is disagreement about which appropriate measures to select [5, 13, 14, 23, 24]. In 2016, the consensus results of an expert panel on metrics assessing the impact of stewardship interventions on a patient level in an acute-care setting were published. Potential metrics were evaluated for four distinct criteria, one of them being the feasibility to monitor the metric in any hospital with an electronic health record. Only six metrics were retained by the expert panel as suitable for ready implementation: incidence of healthcare facility and hospital-onset *Clostridium difficile* infection, rates of antibiotic-resistant pathogens, days of antibiotic therapy/number of admissions, days of antibiotic therapy/patient days and redundant therapy events. All of these metrics may be derived from separate electronic data sources (clinical, pharmacy, microbiology) and a connection between the different elements is not mandatory; however, these metrics are crude and unable to provide insight into antibiotic prescription. By linking these sources, a deeper understanding of the different factors driving antimicrobial use can be obtained, as illustrated by this study.

For example, the respiratory system accounted for half of identified sources of bacterial infection and more than one third of the total antibacterial DOT. Compared to abdominal infections, which represented the second largest group of bacterial infections and destination for antibiotic consumption, respiratory infections were less frequently categorized as highly probable (76% versus 50%, respectively), which also reflected the amount of DOT that was designated to treat these highly probable infections (85% of the total DOT of abdominal and 56% of the total DOT of respiratory infections). One quarter of the infections that were diagnosed in our ICU were ICU-acquired, which is in contrast with the study of Bergmans et al. where half of the infections were ICU-acquired and almost exclusively occurred in ventilated patients. Whereas the authors of the previous study concluded that stewardship should be focused on the prevention of ventilator-associated respiratory infections, this statement may apply less to our ICU population. In addition, restricting duration of antibiotic therapy in VAP of high probability will offer little gain, as the median treatment duration was only 7 days. In contrast, more restrictive use of antibiotics in suspected respiratory tract infections with cultures remaining negative and/or a swift clinical resolution, could result in a more profound reduction of antibiotic consumption. In addition, we observed that prophylactic treatment accounted for one fourth of the total DOT. Although this figure seems extremely high, more in-depth evaluation showed that two thirds of the antibacterial prophylactic treatment consisted of perioperative prophylaxis and prophylaxis in the immunocompromised patient, which conformed to guidelines with regard to treatment duration in the ICU. Whether aspiration and trauma justify antibiotic prophylaxis, and for what duration, is still a matter of debate; more recent studies promote restrictive use of antibiotics in this setting [25–29]. We believe that introduction of more restrictive guidelines on prophylaxis in trauma and aspiration, which are currently unavailable in our ICU, could lead to a reduction in antibiotic use, albeit rather limited if we take the total as well as the median DOT for both indications in our ICU into account. This is in contrast with use of the antifungal prophylactic treatment, which is extremely high in our ICU, particularly in the context of intra-abdominal infections. Clear instructions on prophylactic indication and treatment duration, which are also not in place, may restrain our overall use of antifungals [30].

Despite the recognition of the importance of high-quality surveillance data to support antibiotic stewardship, few studies have provided detailed data on ICU global antibiotic consumption and infection diagnosis over an extended period of time [1, 19, 31]. This may reflect the difficulties in continuous prospective collection of infection-related data due to personnel and time restraints. In fact, while the authors of the study of Bergmans et al. felt that their proposed surveillance model in which they categorize antibiotic indications as prophylaxis, bacteriologically proven infections or non-bacteriologically proven (clinical suspicion) infections, would be suitable for more widespread use, there have since been few publications offering a similarly wide scope on antibiotic use in the ICU [1]. In our ICU, the COSARA software platform facilitated the integration of antibiotic, clinical and microbiological information during the workflow of daily bedside clinical rounds and weekly multidisciplinary staff meetings, hereby illustrating that sustained prospective surveillance is an achievable ambition with the help of information technology [20, 21]. The sustainability of this surveillance is probably to a certain extent due to the rather intuitive approach of labeling infections by the physician choosing from a drop-down menu of possible diagnoses and categorization by infection probability. While the result closely reflects physicians’ judgment and attitude in daily practice, it does not formally adhere to criteria such as those provided by the Centers for Disease Control (CDC) or the European Centre for Disease Prevention and Control (ECDC). A previous analysis assessing the validity of the diagnostic information recorded as such in COSARA compared to conventional surveillance data gathered by using checklists based on CDC-NHSN criteria showed good agreement between both surveillance methods [21]. However, a lack of precision may hamper comparisons between centers (as required for benchmarking) and over time (changing perception). This may to a certain extent be remedied by filtering sets of infection labels for the fulfillment of objective criteria as e.g. presence of positive microbiological cultures, biochemical findings exceeding a given threshold and noting of clinical signs in a computerized medical file.

Our study has limitations. First, building the database starting from computerized physician order entry depends on adequate filling in of the “pop-up” questions that are triggered by it, and by the persistent commitment of attending physicians or infection control personnel in linking the various information sources and finalizing infection diagnosis. Second, as stated before, the lack of adherence to strict criteria in labeling infection diagnosis in the current design hampers multicenter application [21]. Probably a trade-off has to be found between practical feasibility of continued registration on the one hand, and precision in diagnosis on the other. In addition, while COSARA software is compatible with various intensive care information systems, the applicability of our surveillance in different ICU settings and staffing structures has not been formally tested. Therefore, further studies are necessary to validate this model in these settings. Third, COSARA does not capture infections for which no antibiotic treatment is prescribed. Fourth, it remains to be tested to what extent high-quality surveillance may translate into effective stewardship intervening in the treatment decisions of the ICU physician.

## Conclusions

We were able to obtain a unique bird’s eye view on global antibiotic use and infection diagnosis in our ICU over a 4-year time period by analysis of a multifaceted dataset, which was collected during the daily clinical workflow of ICU physicians with the help of information technology. In doing so, we revealed antibiotic prescription patterns that merit the attention of antibiotic stewardship.

## Additional files

Additional file 1: Screenshot of COSARA central infection dashboard view. Time-graphs at the top of the page show the evolution of selected clinical (e.g. fever) and laboratory (e.g. leukocytosis, C-reactive protein (CRP)) variables and indicators of severity-of-illness (e.g. the arterial oxygen tension (PaO_2_)/fractional inspired oxygen (FiO_2_) ratio, sequential organ failure assessment score (SOFA)). For every antibiotic prescription that is entered via computerized physician order entry, a horizontal bar is created that runs just above the timeline and lengthens upon duration of the prescription (antibiotic bar). This bar is accompanied by a second bar running in parallel below the timeline and describing the indication for this antibiotic (infection bar - introduced manually and structured). The infection bar is created in a two-step fashion. A preliminary version is fed by data from a short questionnaire that “pops up” in real time after any antibiotic prescription and inquires the prescriber about indication, likely focus, severity and probability of infection and presence of microbiological data guiding antibiotic choice. This preliminary bar can be altered manually when more data on the origin and clinical evolution of the infection become available. Multiple antibiotic prescriptions can thus be linked with the same infection bar; in addition, the same antibiotic bar can be linked with multiple infection bars (e.g. antibiotic prescribed for simultaneous intra-abdominal and respiratory infection). For each infectious episode, focus, severity and probability of infection is selected from a drop-down menu. More detailed information on prescription and infection are revealed on the base of the screen by hovering over the bars. All positive microbial culture results and matching susceptibility patterns are automatically indicated above the timeline using small symbols, whereas selected microbiological isolates that are linked to an infection are displayed underneath the timeline. (JPG 479 kb)

Additional file 2: Screenshot of COSARA microbiology overview. The results of consecutive microbiological samples that were taken in an individual patient and the corresponding susceptibility patterns are displayed. (JPG 511 kb)

Additional file 3: Screenshot of COSARA antibiotic-infection combinations linked to microbiology data overview. The coupled antibiotic-infection bars can be linked to microbiological culture results; pathogens may be designated as causative pathogens or as non-causative pathogens influencing antibiotic prescription (e.g. nasal carriage of methicillin-resistant *Staphylococcus aureus* promoting glycopeptide prescription in suspected pneumonia with negative sputum cultures). (JPG 298 kb)

Additional file 4: Screenshot of COSARA consecutive chest x-rays view. (JPG 474 kb)

Additional file 5: Patient characteristics. (DOC 36 kb)

Additional file 6: Treatment duration per focus of infection. (DOC 41 kb)

Additional file 7: Antimicrobial use per antimicrobial class and per year. (DOC 61 kb)

## Abbreviations

APACHE II: Acute Physiology and Chronic Health Evaluation II
ASP: Antibiotic stewardship program
CDC-NHSN: Centers for Disease Control and Prevention - National Healthcare Safety Network
COSARA: Computer-based surveillance and alerting of infections, antimicrobial resistance and antibiotic consumption in the ICU
DOT: Days of therapy
ECDC: European Centre for Disease Prevention and Control
ESBL: Extended spectrum beta-lactamase production
ICU: Intensive care unit
IQR: Interquartile range
LOS: Length of stay
VAP: Ventilator-associated pneumonia
VAT: Ventilator-associated tracheobronchitis
