# Supplementary figures and images for: A complete and multifaceted overview of antibiotic use and infection diagnosis in the intensive care unit: results from a prospective four-year registration
Source: Crit Care. 2018 Sep 29;22:241. doi: 10.1186/s13054-018-2178-7 (PMC6162888; doi:10.1186/s13054-018-2178-7)

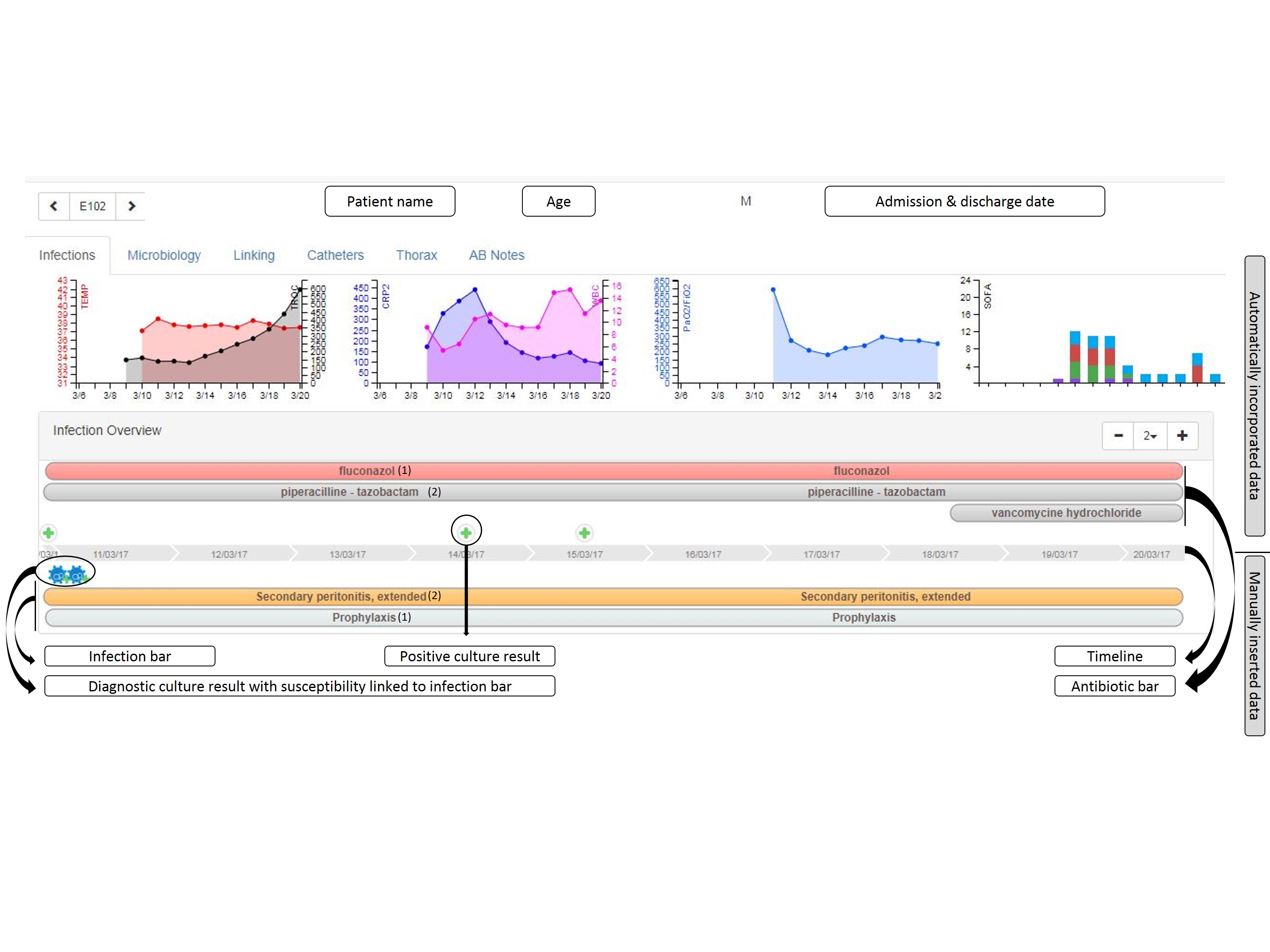

Supplement: Supplementary file 1 — Screenshot of COSARA central infection dashboard view. Time-graphs at the top of the page show the evolution of selected clinical (e.g. fever) and laboratory (e.g. leukocytosis, C-reactive protein (CRP)) variables and indicators of severity-of-illness (e.g. the arterial oxygen tension (PaO2)/fractional inspired oxygen (FiO2) ratio, sequential organ failure assessment score (SOFA)). For every antibiotic prescription that is entered via computerized physician order entry, a horizontal bar is created that runs just above the timeline and lengthens upon duration of the prescription (antibiotic bar). This bar is accompanied by a second bar running in parallel below the timeline and describing the indication for this antibiotic (infection bar - introduced manually and structured). The infection bar is created in a two-step fashion. A preliminary version is fed by data from a short questionnaire that “pops up” in real time after any antibiotic prescription and inquires the prescriber about indication, likely focus, severity and probability of infection and presence of microbiological data guiding antibiotic choice. This preliminary bar can be altered manually when more data on the origin and clinical evolution of the infection become available. Multiple antibiotic prescriptions can thus be linked with the same infection bar; in addition, the same antibiotic bar can be linked with multiple infection bars (e.g. antibiotic prescribed for simultaneous intra-abdominal and respiratory infection). For each infectious episode, focus, severity and probability of infection is selected from a drop-down menu. More detailed information on prescription and infection are revealed on the base of the screen by hovering over the bars. All positive microbial culture results and matching susceptibility patterns are automatically indicated above the timeline using small symbols, whereas selected microbiological isolates that are linked to an infection are displayed underneath the timeline. (JP [file 13054_2018_2178_MOESM1_ESM.jpg]

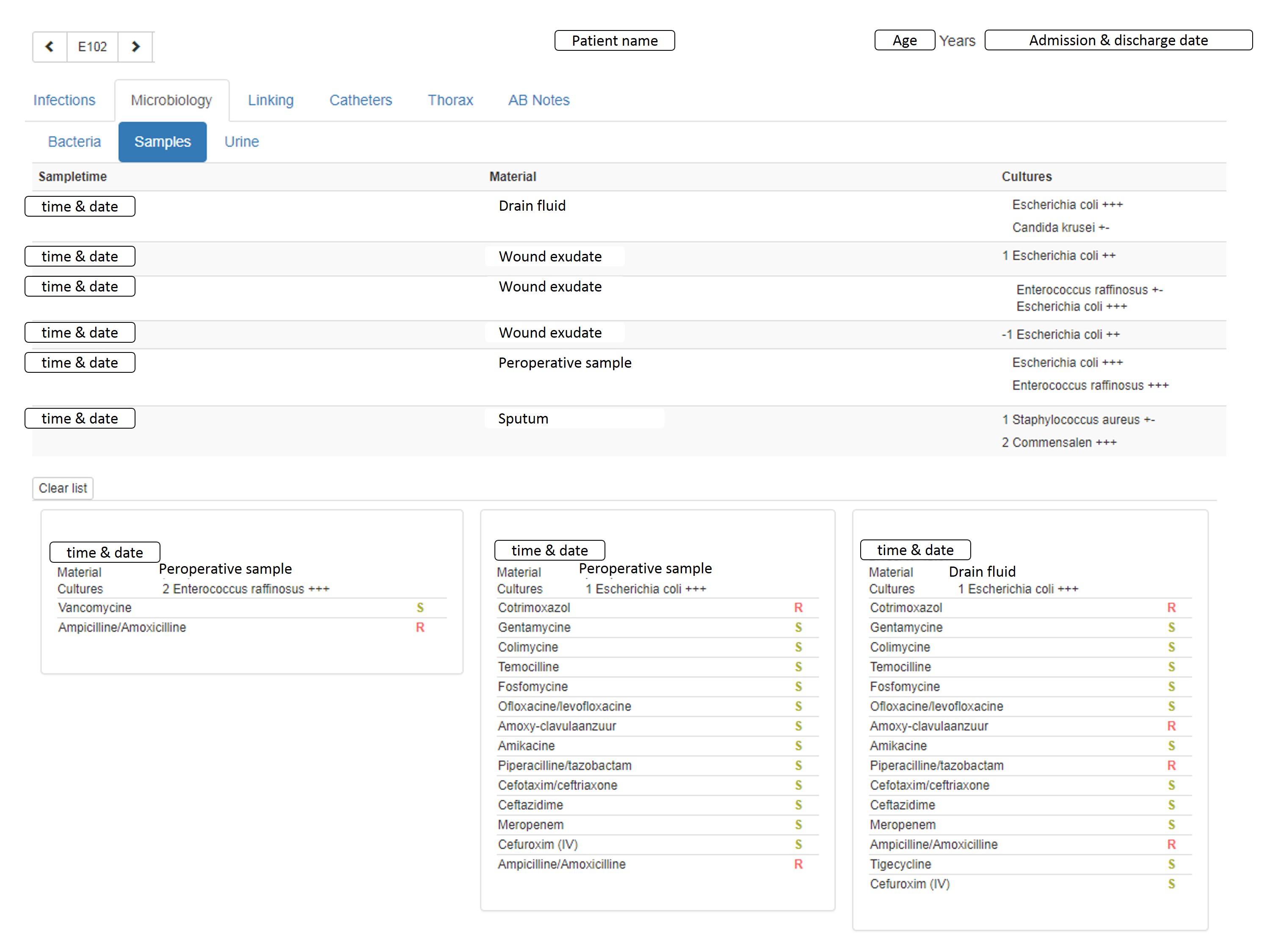

Supplement: Supplementary file 2 — Screenshot of COSARA microbiology overview. The results of consecutive microbiological samples that were taken in an individual patient and the corresponding susceptibility patterns are displayed. (JPG 511 kb) [file 13054_2018_2178_MOESM2_ESM.jpg]

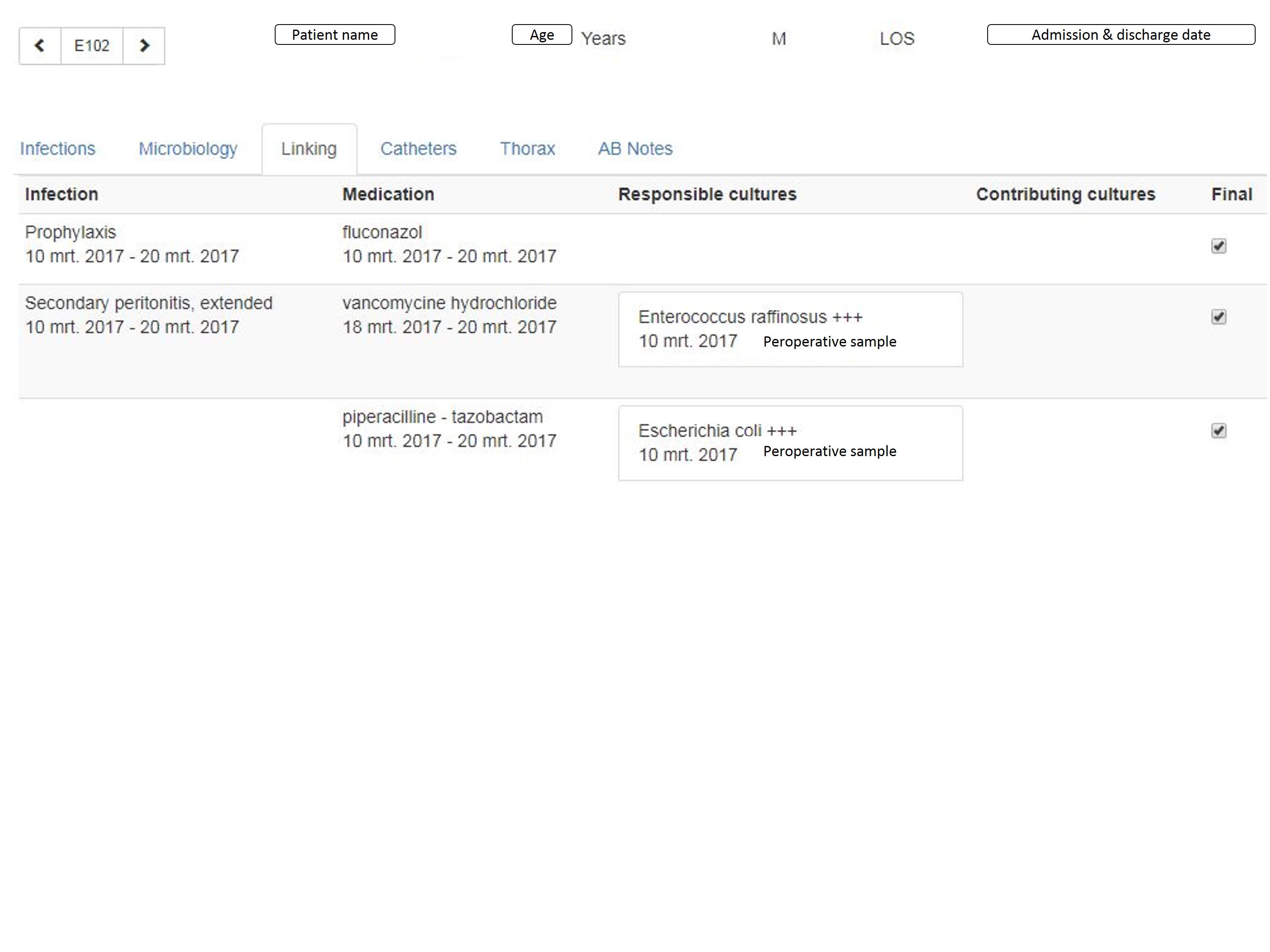

Supplement: Supplementary file 3 — Screenshot of COSARA antibiotic-infection combinations linked to microbiology data overview. The coupled antibiotic-infection bars can be linked to microbiological culture results; pathogens may be designated as causative pathogens or as non-causative pathogens influencing antibiotic prescription (e.g. nasal carriage of methicillin-resistant Staphylococcus aureus promoting glycopeptide prescription in suspected pneumonia with negative sputum cultures). (JPG 298 kb) [file 13054_2018_2178_MOESM3_ESM.jpg]

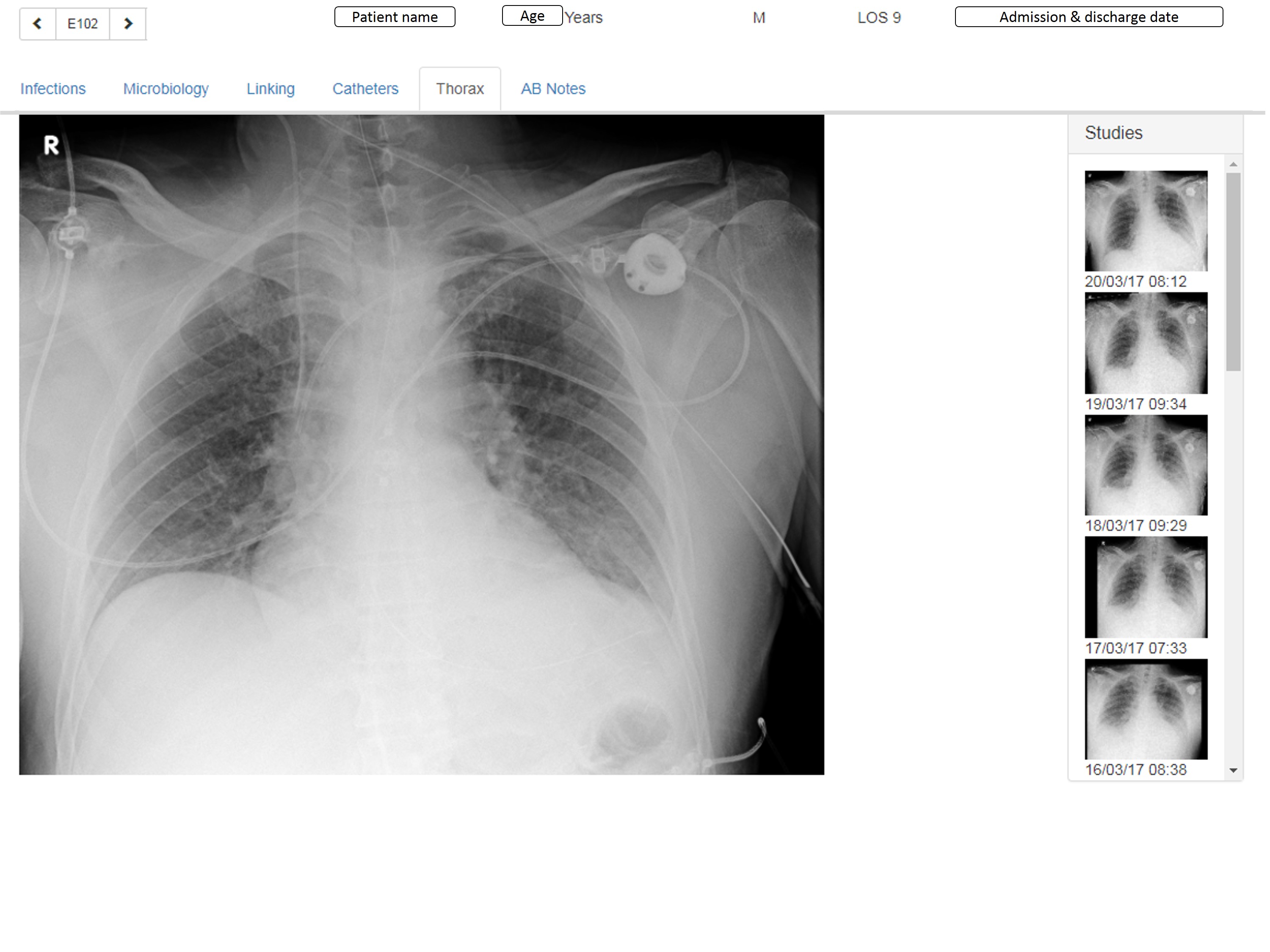

Supplement: Supplementary file 4 — Screenshot of COSARA consecutive chest x-rays view. (JPG 474 kb) [file 13054_2018_2178_MOESM4_ESM.jpg]
