# Supplementary material for: A complete and multifaceted overview of antibiotic use and infection diagnosis in the intensive care unit: results from a prospective four-year registration
Source: Crit Care. 2018 Sep 29;22:241. doi: 10.1186/s13054-018-2178-7 (PMC6162888; doi:10.1186/s13054-018-2178-7)
Supplement: Supplementary file 5 — Patient characteristics. (DOC 36 kb) [file 13054_2018_2178_MOESM5_ESM.doc]

**Additional file 5: Patient characteristics***

|  | LOS ICU <48h (n=4979) | LOS ICU ≥ 48h (n=3784) | *P* value |
| --- | --- | --- | --- |
| Age (years), median (IQR) | 59 [44-70] | 62 [50-72] | <0.001 |
| Male gender, n (%) | 2775 (55.7) | 2401 (63.5) | <0.001 |
| ICU department |  |  | <0.001 |
| Medical ICU, n (%) | 1159 (23.3) | 1552 (41) |  |
| Surgical ICU, n (%) | 3820 (76.7) | 2232 (59) |  |
| Admission type |  |  | <0.001 |
| Urgent, n (%) | 2668 (53.6) | 2676 (70.7) |  |
| Elective, n (%) | 2310 (46.4) | 1108 (29.3) |  |
| APACHE II score, median (IQR) | 15 [11-20] | 22 [16-28] | <0.001 |
| SAPS II score, median (IQR) | 35 [25-51] | 54 [37-72] | <0.001 |
| Mechanical ventilation, n (%) | 1021 (20.5) | 2220 (58.7) | <0.001 |
| Vasopressor therapy, n (%) | 798 (16) | 2147 (56.7) | <0.001 |
| ICU mortality, n (%) | 329 (6.6) | 607 (16) | <0.001 |
| Hospital mortality, n (%) | 447 (9) | 867 (22.9) | <0.001 |

*For outcome analysis, we only included the last ICU episode of patients with consecutive ICU admissions.

LOS = length of stay, ICU = intensive care unit, IQR = interquartile range, APACHE II = Acute Physiology And Chronic Health Evaluation II, SAPS II = Simplified Acute Physiology Score
