# Supplementary material for: A complete and multifaceted overview of antibiotic use and infection diagnosis in the intensive care unit: results from a prospective four-year registration
Source: Crit Care. 2018 Sep 29;22:241. doi: 10.1186/s13054-018-2178-7 (PMC6162888; doi:10.1186/s13054-018-2178-7)
Supplement: Supplementary file 6 — Treatment duration per focus of infection. (DOC 41 kb) [file 13054_2018_2178_MOESM6_ESM.doc]

**Additional file 6: Treatment duration p**er focus of infection

| **Focus of infection** | **All antibiotic courses** | | **Antibiotic course completed on ICU** | |
| --- | --- | --- | --- | --- |
|  | **Number of infections** | **Treatment duration**  **days**  **(median, [IQR])** | **Number of infections** | **Treatment duration**  **days**  **(median, [IQR])** |
| **Respiratory infection** | 2779 | 5 [3-7] | 1389 | 6 [4-9] |
| Community-acquired pneumonia | 281 | 4 [2-6] | 123 | 6 [3.5-8] |
| Aspiration pneumonia | 554 | 5 [3-8] | 293 | 7 [5-9] |
| Hospital-acquired pneumonia | 668 | 5 [3-7] | 279 | 6 [4-8] |
| Healthcare-associated pneumonia | 224 | 4 [2-6] | 81 | 6 [4-8] |
| Tracheobronchitis (not ventilated) | 174 | 4 [2-6] | 83 | 5 [3-7] |
| Ventilation-associated pneumonia | 345 | 7 [5-9] | 264 | 7 [5-10] |
| Tracheobronchitis (ventilated) | 182 | 6 [4-7] | 134 | 6 [4-8] |
|  |  |  |  |  |
| **Abdominal infection** | 1094 | 4 [2-8] | 329 | 8 [4-14] |
| Intra-abdominal collection – abcedation | 201 | 6 [3-12] | 54 | 14.5 [7-20] |
| Localized secondary peritonitis | 155 | 4 [2-9] | 37 | 10 [7-15] |
| Diffuse secondary peritonitis | 137 | 5 [3-10] | 40 | 11.5 [7-18] |
|  |  |  |  |  |
| **Uro-genital infection** | 418 | 3 [2-4] | 158 | 3 [1-6] |
|  |  |  |  |  |
| **Skin & soft tissue infection** | 317 | 4 [2-7] | 82 | 7 [3-11] |
|  |  |  |  |  |
| **CLABSI** | 147 | 4 [2-7] | 66 | 5 [3-9] |
|  |  |  |  |  |
| **Neutropenic fever** | 145 | 4 [2-7] | 42 | 8 [3-9] |

IQR = interquartile range (25^th^ -75^th^ percentile); CLABSI = central-line associated bloodstream infection
