# Supplementary material for: A complete and multifaceted overview of antibiotic use and infection diagnosis in the intensive care unit: results from a prospective four-year registration
Source: Crit Care. 2018 Sep 29;22:241. doi: 10.1186/s13054-018-2178-7 (PMC6162888; doi:10.1186/s13054-018-2178-7)
Supplement: Supplementary file 7 — Antimicrobial use per antimicrobial class and per year. (DOC 61 kb) [file 13054_2018_2178_MOESM7_ESM.doc]

**Additional file 7: Antimicrobial utilization per antimicrobial class and per year**

|  |  | DOT/1000 patient days | | | | |
| --- | --- | --- | --- | --- | --- | --- |
|  | DOT (%) | 2013-2016 | 2013 | 2014 | 2015 | 2016 |
| **Antibacterial class** |  |  |  |  |  |  |
| Aminoglycosides | 474 (1.0) | 10.0 | 8.0 | 13.0 | 8.6 | 10.4 |
| Ansamycins | 268 (0.5) | 5.7 | 6.0 | 6.4 | 6.7 | 3.6 |
| Carbapenems | 4488 (9.1) | 94.7 | 102.6 | 90.0 | 90.8 | 95.0 |
| 1st gen. cephalosporins | 2939 (6.0) | 62.0 | 56.8 | 63.5 | 61.1 | 66.8 |
| 2nd gen. cephalosporins | 1192 (2.4) | 25.1 | 36.0 | 28.8 | 20.2 | 15.1 |
| 3rd gen. cephalosporins | 1955 (4.0) | 41.2 | 41.0 | 37.0 | 39.5 | 47.4 |
| Fluoroquinolones | 5385 (11) | 113.6 | 116.9 | 120.5 | 94.8 | 121.5 |
| Folate pathway inhibitor | 3105 (6.3) | 65.5 | 76.8 | 68.4 | 55.9 | 60.2 |
| Glycopeptides | 2966 (6.0) | 62.6 | 66.8 | 61.1 | 67.1 | 55.3 |
| Glycylcyclines | 319 (0.6) | 6.7 | 6.7 | 4.2 | 7.2 | 8.9 |
| Lincosamides | 806 (1.6) | 17.0 | 17.3 | 15.2 | 15.2 | 20.3 |
| Macrolides | 1421 (2.9) | 30.0 | 18.2 | 29.7 | 37.0 | 35.5 |
| Monobactams | 150 (0.3) | 3.2 | 3.1 | 3.5 | 3.9 | 2.1 |
| Nitrofurans | 59 (0.1) | 1.2 | 1.7 | 1.4 | 1.0 | 0.8 |
| Nitroimidazoles | 1289 (2.6) | 27.2 | 26.9 | 26.3 | 28.4 | 27.1 |
| Oxazolidinones | 1780 (3.6) | 37.6 | 32.6 | 38.6 | 35.5 | 43.6 |
| Penicillins | 1504 (3.1) | 31.7 | 33.0 | 26.0 | 24.2 | 43.6 |
| Non-anti-pseudomonal penicillins + beta-lactamase inhibitor | 8136 (16.5) | 171.6 | 176.3 | 161.7 | 179.4 | 169.4 |
| Anti-pseudomonal penicillins + beta-lactamase inhibitor | 10342 (21.0) | 218.2 | 215.8 | 219.3 | 220.5 | 217.2 |
| Phosphonic acids | 27 (0.1) | 0.6 | - | - | 0.9 | 1.4 |
| Polymyxins | 469 (1.0) | 9.9 | 12.1 | 14.3 | 7.0 | 5.9 |
| Tetracyclines | 95 (0.2) | 2.0 | - | 0.7 | 7.5 | - |
| Total antibacterial | 49169 (100) | 1037.3 | 1054.8 | 1029.6 | 1012.5 | 1051.2 |
|  |  |  |  |  |  |  |
| **Antifungal class** |  |  |  |  |  |  |
| Azoles | 7684 (83.1) | 162.1 | 151.2 | 176.4 | 159.2 | 161.7 |
| Echinocandins | 1354 (14.6) | 28.6 | 29.3 | 19.2 | 35.7 | 30.3 |
| Polyenes | 206 (2.2) | 4.3 | 3.3 | 6.3 | 3.3 | 4.5 |
| Total antifungal | 9244 (100) | 195.0 | 183.8 | 201.9 | 198.2 | 196.5 |
|  |  |  |  |  |  |  |
| **TOTAL** | **58413**  **(100)** | **1232.3** | **1238.6** | **1231.4** | **1210.7** | **1247.7** |

DOT, days of therapy
